# Spontaneous Splenic Rupture as a Complication of Malaria and Incidental Acute Appendicitis: A Case Report

**DOI:** 10.7759/cureus.19028

**Published:** 2021-10-25

**Authors:** Ahmad Odeh, Abdulmohsen Alsuwaigh, Abdulqader M Albeladi, Zaki Busbaih, Abdullah M Alkhars, Mohammed O Khalid, Aminah H AlAli, Mohmmed T AlAbbad, Kawthar A Boumarah, Hussain M Alkhars, Zainab A Alammar, Ahmed H Almohammedsaleh

**Affiliations:** 1 General Surgery, Prince Saud Bin Jalawi Hospital, Al Ahsa, SAU; 2 General Surgery and Laparoscopy, Prince Saud Bin Jalawi Hospital, Al Ahsa, SAU; 3 Orthopaedics, King Fahad Hospital Hofuf, Al Ahsa, SAU; 4 General Surgery, King Fahad University Hospital, Khobar, SAU; 5 Medicine, George Washington University, Washington, USA; 6 Medicine, King Faisal University, Al Ahsa, SAU

**Keywords:** appendectomy, splenectomy, appendicitis, malaria, complication, spontaneous splenic rupture

## Abstract

Malaria presents a challenge to healthcare systems globally. It is associated with severe complications, notably splenic rupture. The prognosis of malaria complicated by splenic rupture is poor and sometimes leads to death even with timely intervention. Here, we report the case of a patient who presented with complicated malaria with spontaneous splenic rupture and coincidental acute appendicitis. A 34-year-old man was successfully treated for a grade IV ruptured spleen and acute appendicitis with splenectomy and appendectomy. Postoperative care took place in the intensive care unit and the patient was shifted to the general floor on the fifth day. Upon discharge the next day, he was prescribed amoxicillin twice daily for one year. Malaria can present with life-threatening complications requiring prompt surgical intervention.

## Introduction

Malaria has been affecting health globally, with an impact greater than any other infectious disease. More than 500 million individuals are infected worldwide and more than 2.5 million die every year [1]. Severe forms of malaria can lead to severe complications such as shock, respiratory distress, severe anemia, multiple convulsions, intra-abdominal organ inflammation, and bleeding caused by spontaneous splenic rupture [2].

The most common causes of splenic rupture are trauma, hematological malignancies, and infections. However, in malaria-endemic regions, infectious causes of splenic rupture outnumber hematological malignancies. Regardless of early detection and good management plans, the mortality rate in spontaneous splenic rupture cases can be up to 38% of affected individuals [3]. Even though malaria can be hidden as an asymptomatic condition, it can present at the first visit as a case of acute abdomen. The presentation of malaria can be more complex and mimic several other conditions [2,4].

Here, we report an unusual case of malaria complicated by spontaneous splenic rupture and acute appendicitis which raises concerns over rare, but possible, severe malaria complications and their impact on the prognosis of patients.

## Case presentation

A 34-year-old Sudanese male was admitted to Prince Saud Bin Jalawi Hospital, Al Ahsa, Saudi Arabia as a case of Malaria. As the patient complained of fever with no clear cause by routine investigations, he was admitted as a case of fever of unknown origin. During admission, the patient was diagnosed with typhoid using the Widal test. However, over two to three days, the pattern of fever was not characteristic of typhoid as the patient had recently arrived from Sudan. Hence, a malaria test was performed which was positive. For malaria, the patient was started on ciprofloxacin, quinine sulfate, and doxycycline. After initiating the treatment, his fever improved and he was ready for discharge.

On the third day of the admission, the patient developed generalized abdominal pain associated with nausea, inability to pass flatus or stool for 24 hours, and high-grade fever. On examination, he looked pale, severely ill, his temperature was 39°C, his pulse rate was 120 beats per minute, his respiratory rate was 34 breaths per minute, and his blood pressure was 80/65 mmHg. An abdominal examination revealed a rigid abdomen with tenderness all over but more pronounced in the left hypochondriac region and the right iliac fossa with absent bowel sounds.

Resuscitation was started immediately as he was deemed critically ill and a candidate for immediate surgical intervention. A sepsis survival campaign was initiated with an oxygen mask. Additionally, two wide bore cannulas were placed and a blood sample was obtained for laboratory investigations. Four units of packed red blood cells were prepared, and broad-spectrum antibiotics along with a bolus of pre-warmed crystalloid solution were administered.

**Figure 1:**
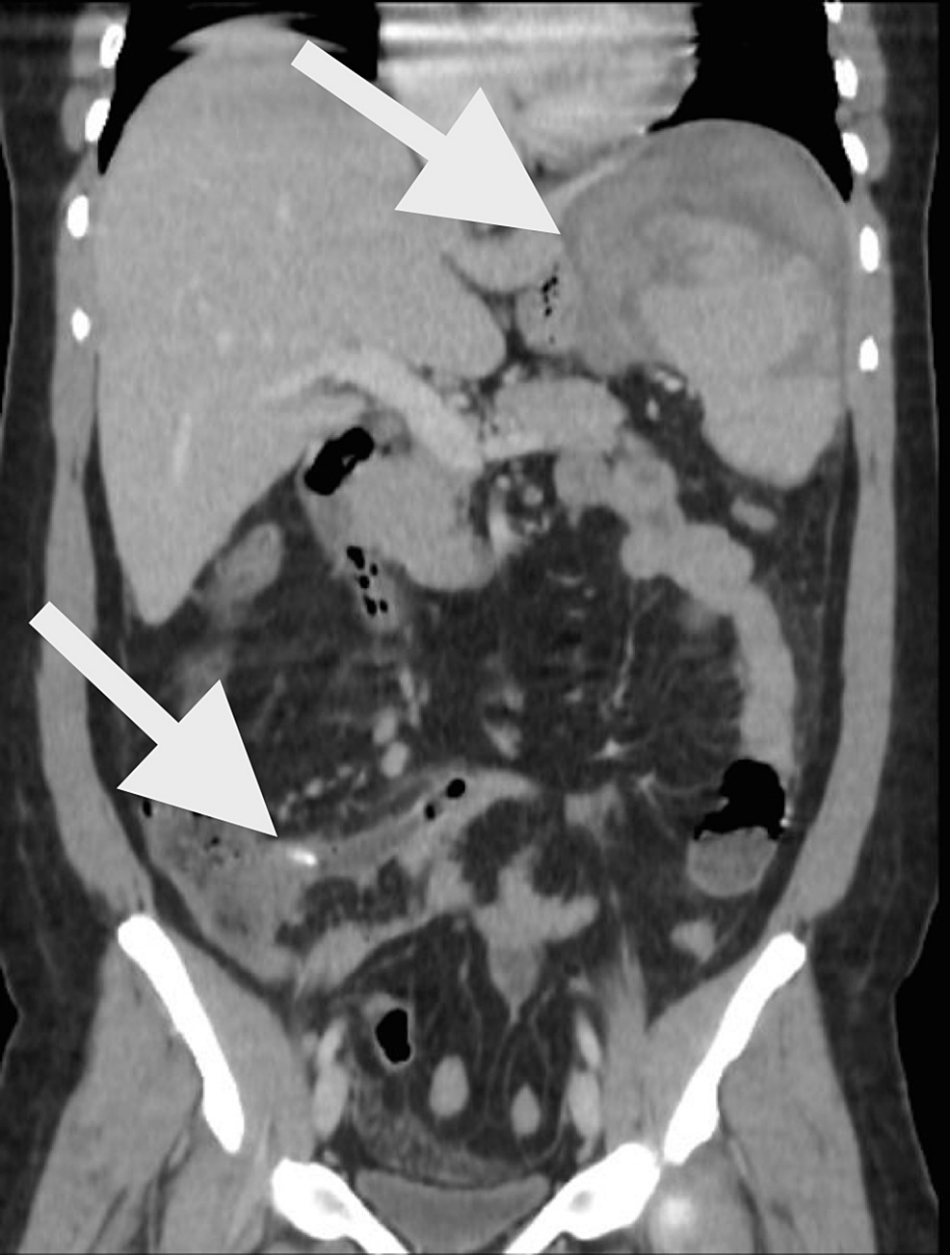
Abdominopelvic CT scan findings. The first arrow shows the grade IV spontaneously ruptured spleen. The second arrow shows the dilated appendix (15 mm) with fecalith, representing acute appendicitis.

After stabilizing the patient, an abdominopelvic CT scan was performed which showed a grade IV ruptured spleen and acute appendicitis (Figure 1). The findings were explained to the patient and his consent was obtained for splenectomy and an appendectomy.

Under general anesthesia, the patient underwent a midline laparotomy. Four-quadrant abdominal packing was done after the removal of clots and suction of blood. An emergency splenectomy was performed by dividing the splenic ligament and delivering the spleen to the surgical field where short gastric vessels followed by splenic artery and vein were ligated and divided. Following the splenectomy, an appendectomy was also performed. The appendix was inflamed and contained fecalith. The abdominal cavity was washed out with a copious amount of pre-warmed crystalloid solution. Two drains were placed: one at the splenic bed and another at the pelvis. PDS 0 was used for mass closure of the abdomen.

Histological analysis of the spleen specimen and appendix showed malaria merozoite-like bodies in addition to the foci of infarcted necrosis, which confirmed malaria and acute suppurative appendicitis and serositis.

The patient was then transferred to the intensive care unit where he was kept under strict monitoring of vital signs, blood chemistries, and input-output chart of the nasogastric tube, drains, and Foley catheter. After two days, the nasogastric tube and Foley catheter were removed, where both the splenectomy drain and appendectomy drain were removed on the fourth day. He had a good recovery, and, on the fifth day, he was shifted to the general floor where anti-malaria medications were restarted.

Table 1 lists the results of the laboratory investigations done at the time of discharge. The patient received pneumococcal vaccination, *Haemophilus influenzae* type b vaccination, meningococcal vaccination, and was prescribed amoxicillin 250 mg per oral twice daily for one year.

**Table 1:** Results of the laboratory investigations.

Laboratory test	Result	Reference range
White blood cells	14.1	4.5–11 × 10^9^/L
Hemoglobin	10.7	13.8–17.2 g/dL
Platelets	650	150–450 × 10^3^/µL
Albumin	42.5	34–54 g/L
Alkaline phosphatase	84	44–147 IU/L
Alanine aminotransferase	35	7–55 U/L
Aspartate aminotransferase	21	5–40 U/L
Total bilirubin	5.2	5.1–17 µmol/L
Direct bilirubin	1.11	1.7–5.1 µmol/L
Total protein	89.2	60–83 g/L
Fasting blood glucose	5.8	3.9–5.6 mmol/L
Potassium	4.72	3.5–5 mEq/L
Sodium	133	135–145 mEq/L

## Discussion

*Plasmodium vivax* is the most widely spread human malaria parasite, with 2.5 billion people at risk [5]. It caused 8.5 million infections in 2015, with 58% occurring in the Southeast Asia region and causing 3,000 deaths. *P. vivax* infection can result in severe anemia, hepatic dysfunction, and jaundice, as well as acute lung injury, acute respiratory distress syndrome, pulmonary edema, shock, acute renal failure, severe thrombocytopenia, and splenic rupture [6,7]. Multiple organ failure and severe life-threatening conditions are also associated.

The spleen aids in the host response against *Plasmodium* and other intravascular parasites. Splenic structure and function are altered during acute and chronic malaria, resulting in mild-to-severe enlargement and complications. The underlying pathophysiology of spleen rupture is still being unraveled. However, two hypotheses have been proposed to explain the rupture, both of which argue that it occurs in the acute form of malaria. The first suggests that malformed infected red blood cells with altered surface properties activate lymphatic tissue in the spleen and cause significant stasis in its sinuses leading to its rupture [8]. In the second hypothesis, the rupture of the spleen is proposed to be caused by external compression by the abdominal musculature during physiological actions such as sneezing, coughing, defecation, and sitting up or turning in bed [9].

Regarding diagnosis and clinical implications, it is vital to remember that the longer the symptoms of malaria linger, the greater the possibility of splenic rupture, making early identification critical for prevention [10]. In that regard, splenic rupture should always be suspected in a patient from an endemic region who develops hypotension and discomfort in the left hypochondrium acutely [9]. Ultrasonography, CT, and arteriography can be used to confirm a splenic rupture in a hemodynamically stable patient. CT has supplanted angiography as the standard diagnostic method and can be used to diagnose and monitor patients who are being treated conservatively for splenic rupture [9]. However, in many endemic regions, CT is neither cost-effective nor readily available. Ultrasound, on the other hand, can be more economically and geographically accessible. A portable ultrasound utilized promptly by a skilled practitioner can save many lives.

Non-operative management can be employed in carefully selected cases [10,11]. In certain cases of non-traumatic rupture, transcatheter splenic artery embolization can be performed [12]. Splenectomy is still the treatment of choice in life-threatening situations with uncontrolled bleeding and hemorrhagic shock [11]. Regarding operative management, spleen-preserving procedures should be the gold standard wherever possible, especially in patients with a high risk of future malaria exposure [13-16]. In our case, non-operative management was not an option because the patient had coincidental appendicitis with fecalith. In addition, the spleen was completely shattered, further supporting the need for operative management.

## Conclusions

The case presented here illustrates a rare but well-documented malaria complication. The patient had coincidental appendicitis with fecalith and his spleen was completely shattered, which necessitated surgical intervention. Although splenic rupture due to severe malaria is uncommon, it is likely underdiagnosed and underreported. It is a potentially fatal malaria complication that can occur even after starting medical treatment. Consequently, early detection and proper disease care are critical. Therefore, a multidisciplinary approach is advised. In selected, properly monitored patients, conservative treatment should be considered. However, in life-threatening conditions involving uncontrolled bleeding and hemorrhagic shock, splenectomy remains the treatment of choice.
